# Blockade of Cholecystokinin Type 2 Receptors Prevents the Onset of Vincristine-Induced Neuropathy in Mice

**DOI:** 10.3390/pharmaceutics14122823

**Published:** 2022-12-16

**Authors:** Amandine Bernard, Aurore Danigo, Mohamad Mroué, Amandine Rovini, Laurence Richard, Angélique Nizou, Alexis Desmoulière, Franck Sturtz, Claire Demiot, Sylvie Bourthoumieu

**Affiliations:** 1 NeurIT Neuropathies et Innovations Thérapeutiques UR 20218, Faculty of Medicine and Pharmacy, University of Limoges, 87025 Limoges, France; 2 Department of Neurology, Reference Center for Rare Peripheral Neuropathies, University Hospital of Limoges, 87042 Limoges, France; 3 Department of Biochemistry and Molecular Genetics, University Hospital of Limoges, 87042 Limoges, France; 4 Department of Cytogenetics, Medical Genetic and Reproduction Biology, University Hospital of Limoges, 87042 Limoges, France

**Keywords:** chemotherapy-induced peripheral neuropathy, vincristine, allodynia, proglumide, Ly225910, CCK2R

## Abstract

Vincristine (VCR) is responsible for the onset of the VCR-induced peripheral neuropathy (VIPN), associated with neuropathic pain. Several reports have strongly linked the cholecystokinin type 2 receptor (CCK2R) to nociceptive modulation. Thus, our aim was to evaluate the effect of CCK2R blockade on the onset of VIPN, as well as its interaction on VCR anticancer efficacy. VCR was administrated in mice for 8 days (100 µg/kg/d, i.p.). Transcriptomic analysis of the dorsal root ganglia (DRG) was performed at day 7 in VCR and control mice. Proglumide (30 mg/kg/d), a CCK1R and CCK2R antagonist, and Ly225910 (1 mg/kg/d), a selective CCK2R antagonist, were administrated one day before and during VCR treatment. Tactile sensitivity was assessed during treatments. Immunofluorescence and morphological analyses were performed on the skin, DRG and sciatic nerve at day 7. The cytotoxicity of VCR in combination with proglumide/Ly225910 was evaluated in human cancer cell lines. *Cck2r* was highly upregulated in the DRG of VCR mice. Proglumide accelerated the recovery of normal sensitivity, while Ly225910 totally prevented the onset of allodynia and nerve injuries induced by VCR. Proglumide or Ly225910 in combination with VCR did not affect the cytotoxicity of VCR. Targeting CCK2R could therefore be an effective strategy to prevent the onset of VIPN.

## 1. Introduction

Vincristine (VCR) belongs to the family of vinca alkaloids and is one of the most common and effective chemotherapeutic drugs for treating of a broad range of cancers. VCR is also the most neurotoxic agent of its drug class and is responsible for the onset of VCR-induced peripheral neuropathy (VIPN) [1]. Similar to other anticancer agents, VCR shows limited passage across the blood–brain barrier [2], but does accumulate in the dorsal root ganglia (DRG) and peripheral nerves, which has been suggested to cause peripheral neurotoxicity [3,4]. The VIPN is a sensory length-dependent axonopathy, accompanied by neuropathic pain such as paresthesia and allodynia [5]. The onset of VIPN-associated pain necessitates the discontinuation of treatment, significantly influencing the survival of cancer patients and negatively affecting their quality of life. This pathology affects between 30 and 40% of patients and therefore represents a significant public health issue [6]. The only presently accepted preventive measures to limit the onset of VIPN are screening for pre-existing neuropathies and the early detection of clinical signs of neuropathy in subjects receiving neurotoxic chemotherapy [7]. No preventive therapies have thus far shown significant clinical efficacy [8], leaving the search for biomarkers and new targets involved in chemotherapy-induced neuropathies an active and open research field. However, the exact pathophysiological mechanisms of VCR-induced neuropathic pain remain unclear, significantly hampering the development of effective therapeutic strategies that could either prevent or alleviate the symptoms of VIPN. 

Recent reports have reviewed the strongly link between CCK2R and the nociceptive process [9,10]. CCK2R is a G-protein-coupled receptor well known for its roles in satiety and anxiety [11,12]. CCK2R is expressed in the gastrointestinal system as well as in areas of the brain involved in pain modulation and other processes such as memory, anxiety and thermoregulation. Several studies have confirmed that the CCK2R acts as a neuromodulator in sensation and pain tracts through interaction with dopamine, glutamate and other neuropeptides involved in pain regulation [12,13,14]. Upregulation of CCK2R in rodent DRG has been found in a model of burn-induced pain using transcriptomic analysis [15], as well as in other models of neuropathic pain associated with nerve injury [16,17]. In this study, we thus sought to elucidate the contribution of CCK2R to VCR-induced pain and to examine if the inhibition of CCK2R may be a potentially effective strategy to prevent the development of VCR-induced neuropathic pain. 

To further explore the mechanisms underlying VIPN, in this study, we performed a transcriptomic analysis on mouse DRG after VCR exposure. Given that CCK2R was found among the differentially expressed genes, we investigated its area of distribution in the mouse peripheral nervous system. Then, we explored the effect of CCK2R blockade by two pharmacological tools (i.e., proglumide and Ly225910) on the onset of VIPN and maintenance of VCR efficiency as an anticancer agent on human cancer cell lines. Our study may provide insights into understanding the painful mechanisms of VIPN by highlighting the relevance of CCK2R as a promising target to be considered for VIPN therapy.

## 2. Materials and Methods

### 2.1. Animals

This study conformed to the European Community guidelines for the ethical care of experimental animals and was approved by the French Ministry of Higher Education and Research (APAFiS # 27947-2020111216498126 v2). Animal experiments are reported in compliance with ARRIVE guidelines [18]. All effort was made to limit suffering and the number of animals used in the following experiments. A total of 92 male and female (45:45) Swiss mice (6–7 weeks old) from Janvier labs (Saint Berthevin, France) were housed in groups of 4–5 per cage and maintained on a 12 h light/dark cycle with food and water available ad libitum (BISCEm-animal care and facility center). Shredded paper nesting material was supplied for environmental enrichment. 

### 2.2. Transcriptomic Study

#### 2.2.1. Tissue Collection

Twelve mice were randomly assigned in two groups of 6 each, with a sex ratio of 1:1. One group received a daily injection of VCR (100 µg/kg) over 8 days (VCR group), and the other received an equal volume of saline solution (Ctrl group). The day of the last injection, animals from the VCR and Ctrl groups were sacrificed by cervical dislocation after isoflurane anesthesia (3%). The spinal cord was dissected, and ten DRG and two sciatic nerves per mice were removed. Six DRG were dedicated to mRNA extraction (cf. Section 2.3.2), and the four DRG of the L4–L5 lumbar level and the sciatic nerves were dedicated to an immunofluorescence study on CCK2R (cf. Section 2.3.5).

#### 2.2.2. mRNA Extraction

DRG were crushed and mRNA was extracted using the miRNeasy Mini kit (Qiagen, Hilden, Germany), according to the manufacturer’s instructions. Analysis of the quality and quantity of mRNA was performed by Bioanalyzer 2100 (Agilent technologies, Les Ulis, France). Only samples with an RNA integrity number (RIN) up to 7 were loaded on the gene expression microarray.

#### 2.2.3. Transcriptomic Analysis

Transcriptomic analysis was performed using an expression chip, namely SurePrint G3 Mouse Expression v2 (Agilent technologies). Samples were processed following the manufacturer’s instructions. After 17 h of hybridization at 65 °C, the chip was washed several times and then placed in the SureScan DX MicroArray (Agilent technologies). Analysis of the chip data was carried out using Genespring. Fold Change (FC), and *p*-values were obtained after data normalization and *t*-test analysis comparing the Ctrl and VCR groups. Limits were applied to FC > 1.5 and *p*-value < 0.01. 

Gene ontology (GO) analysis was conducted using the online tool database for Annotation, Visualization and Integration Discovery (DAVID, http://david.abcc.ncifcrf.gov/, accessed on 16 April 2020). GO enrichment analysis is used to annotate genes and to find GO terms which are over- or under-expressed using annotations for a specific gene set. GO enrichment analysis was divided into 3 parts: biological process (BP), cell component (CC) and molecular function (MF). A first global analysis was performed including all differentially expressed genes (DEG), highlighted using the Genesprings software (FC < 1.5 and *p*-value < 0.01). Then, two sub analyses were processed on the set of upregulated gene and on the set of downregulated ones. Limits were applied to an enrichment score > 0.5 and *p* value < 0.05.

#### 2.2.4. RT-qPCR

mRNA was transcripted to cDNA using the QuantiTect Reverse Transcription Kit (Qiagen), according to the manufacturer’s instructions. Subsequently, cDNA was subjected to real-time PCR using Rotor-Gene SYBR^®^ Green PCR Kit (Qiagen). The variation of expression for each gene (primer sequences, Table 1) was calculated using the ΔCt method, with the house-keeping gene *Hprt* as an internal control.

#### 2.2.5. Localization of CCK2R in the Sensory Nervous System

Four lumbar (L4–L5) DRG and the two sciatic nerves per mouse (n = 6 in each group, Ctrl and VCR) were collected, post-fixed overnight in 4% paraformaldehyde (PFA), cryoprotected (30% sucrose) and then frozen at −80 °C. DRG sections were cut at 8 µm and incubated overnight with antibody against CCK2R (goat polyclonal, 1:200, Abcam, Cambridge, UK). Sciatic nerve sections were incubated with antibodies against CCK2R and S100 (rabbit polyclonal, 1:500, Dako-Omnis Agilent, Santa Clara, CA, USA) or MBP (mouse monoclonal, 1:500, BioLegend, Amsterdam, The Netherlands). Sections were then incubated with the appropriate secondary antibodies. 

### 2.3. Pharmacological Study

#### 2.3.1. Chemicals

The anticancer agent, VCR, was obtained from the Hospital Pharmacy of Limoges (vincristine Hospira^®^, 2 mg/2 mL, Pfizer, New York, NY, USA) and diluted in saline solution (NaCl 0.9% in water for injection). Proglumide sodium salt (purity > 99%) was purchased from Sigma (Sigma-Aldrich Chimie, Saint-Quentin-Fallavier, France) and dissolved in 10% dimethyl sulfoxide (DMSO) in saline solution. Ly225910 (purity > 99%) was purchased from Abcam (Abcam (Netherlands) B.V., Amsterdam, The Netherlands) and dissolved in 10% DMSO in saline solution.

#### 2.3.2. Schedule Treatments

**Figure 1:**
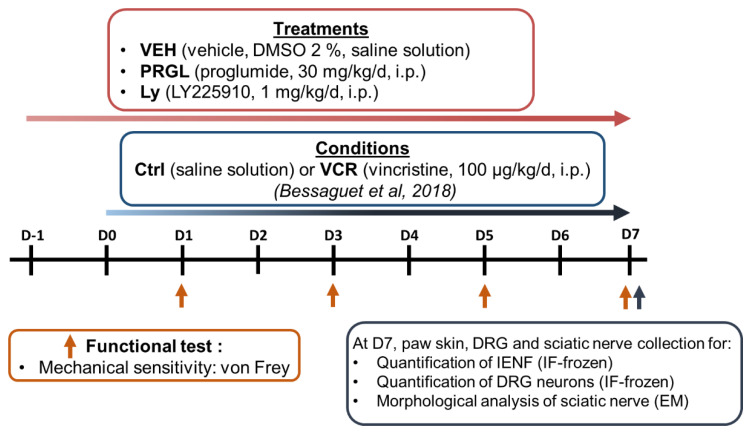
Schematic representation of the pharmacological study design [19]. CCK2R: cholecystokinin type 2 receptor; Ctrl: control, DMSO: dimethyl sulfoxide, DRG: dorsal root ganglion, EM: electron microscopy, IF: immunofluorescence, i.p.: intraperitoneal, Ly: Ly225910, PRGL: proglumide, VEH: vehicle, VCR: vincristine.

Peripheral neuropathy was induced by a daily injection over 8 days of VCR (100 µg/kg/day, intraperitoneally (i.p.), VCR groups) [19] or an equivalent volume of saline solution, i.p. (Ctrl groups (Figure 1)). Preventive treatments started one day prior to the first VCR administration and were administrated each day for 8 days. Proglumide (30 mg/kg/day, i.p.) and Ly225910 (1 mg/kg/day, i.p.) were diluted in a final solution of 2% DMSO (VEH). VEH-treated mice received injections of an equivalent volume of 2% DMSO. The proglumide dose used has previously been shown to have a neuroprotective effect in rodent models of spinal cord injury [15]. The dose of Ly225910 was chosen based on a study showing the beneficial effects of Ly225910 in a stress model [20]. Injections of vehicle, proglumide or Ly225910 were performed several hours before VCR or saline (Ctrl) administration on the days of coadministration. Thus, pharmacological analysis was performed on 6 groups of mice (n = 10 in each group, sex ratio 1:1):

- Ctrl-VEH
- Ctrl-PRGL
- Ctrl-Ly
- VCR-VEH
- VCR-PRGL
- VCR-Ly

Mice were assigned to each group using an online randomization tool (http://www.graphpad.com/quickcalcs/index.cfm, accessed on 7 October 2020).

Assessment of tactile sensitivity was performed on days 1, 3, 5 and 7 in all groups using the Von Frey filament test. Paw skin, DRG and sciatic nerve were removed on six animals per group at the end of experiment (D7) for subsequent immunofluorescence and morphological analyses (Figure 1).

#### 2.3.3. Von Frey Filament Test

Tactile sensitivity was assessed using Von Frey filaments (Bioseb, Vitrolles, France) [21]. The behavioral test was assessed by the same researcher, blinded to the treatment (VEH, PRGL or Ly) and the conditions (Ctrl or VCR). Animals were acclimatized to the testing room for at least 1 h prior to behavioral testing on each day. Then, mice were placed in a plastic cage with a wire mesh floor, which allowed access to their paws, for a resting time of 30 min. The plastic cage was covered to avoid visual stimulation. The area tested was the mid-plantar left hind paw. The mechanical threshold was tested using a modification of the simplified up-down method [22]. Each test round started with filament #6 (0.40 g) and progressed to higher or lower filament values depending on the animal’s response. Each animal went through three test rounds for each paw for each experimental condition. The mechanical threshold was expressed in grams (g). 

#### 2.3.4. Quantification of Intraepidermal Nerve Fiber (IENF) and DRG Neuron Densities

To assess sensory innervation, animals (n = 6 per group) were sacrificed at D7 by cervical dislocation following isoflurane anesthesia. Footpads were then removed using a 3 mm punch biopsy, fixed overnight in 4% PFA, cryoprotected (30% sucrose) and frozen at −20 °C. Sections were cut on a cryostat at 20 µm and incubated overnight with a primary antibody directed against Protein gene product 9.5 (PGP9.5, rabbit monoclonal, 1:50; Abcam). Sections were then incubated with a secondary antibody conjugate to Cy3 (1:500; Jackson Immunoresearch, Suffolk, UK). Epidermal nerve fibers were counted blindly at 400× magnification (Eclipse 50i, Nikon Europe B.V., Amstelveen, The Netherlands), according to established guidelines for human studies [23]. The length of the dermo-epidermal junction was determined using NIS-Elements BR 2.30 software (Laboratory Imaging s.r.o, Nikon, corporation, Prague, Czech Republic) and defined as the epidermal length. Epidermal nerve density was defined as the number of epidermal nerves divided by the epidermal length (IENF number/mm). Three slides per mouse were counted.

To assess the density of DRG neurons, four lumbar (L4–L5) DRG per mice were collected and processed as described above, except that 8 µm sections were sampled. Each DRG section was photographed at 200× using fluorescence microscopy in a systematic fashion. Immunoreactive DRG neurons were counted, and only the area containing neurons was measured using NIS-Elements BR2.30 software (Laboratory Imaging s.r.o, Nikon, corporation, Prague, Czech Republic). The density of PGP9.5^+^ neurons was expressed as neurons/mm^2^. Three sections per DRG were counted [24].

#### 2.3.5. Sciatic Nerve Ultrastructural Analysis

To assess the morphology and to quantify myelinated nerve fibers, sciatic nerves were dissected, placed in 2.5% glutaraldehyde solution diluted in Sorensen buffer, dehydrated and embedded in Epon 812 resin (Euromedex, Souffelweyersheim, France). Semi-thin sections were stained with toluidine blue. Ultrathin sections were stained with uranyl acetate and lead citrate and observed by electron microscopy (JEM-1400 Flash, Jeol, Peabody, MA, USA). Six photographs per animal (n = 6 per group), covering the entire section of sciatic nerve, were taken at 3000× magnification, and the number of myelinated fibers per mm^2^ was counted to calculate the density [24].

### 2.4. Evaluation of Cancer Cell Viability

SU-DHL-4 (ATCC^®^ CRL-2957™) cells were seeded in 96-well plates at 2 × 10^4^ cells/well, and U-2932 (DSMZ^®^ ACC633) at 1 × 10^4^ cells/well. After incubation for 24 h, cells were treated with Ly225910 or proglumide alone at doses ranging from 0 to 10 nM or from 0 to 5 mM, or in combination with VCR at the half-maximal inhibitory concentration (IC50) for 24 h. Then, 20 µL of thiazolyl blue tetrazolium bromide (MTT) solution at 5 mg/mL were added to the 100 μL of medium in each well and then incubated with the cells for 3 h. After incubation, the medium was removed by centrifugation at 600 rpm for 10 min, and 150 μL of DMSO was added to each well to solubilize the formazan crystals. Absorbance was measured at 490 nm using a microplate reader (Lab Systems, MultiskanEX, Waltham, MA, USA) [25]. Cell viability was expressed as the percentage of MTT reduction, assigning a 100% value to the absorbance value of untreated cells. All experiments were performed three times. The results are presented as the mean ± standard error of mean (SEM).

### 2.5. Data Analysis

All data were analyzed using the GraphPad Prism 8 software and expressed as the mean ± SEM. A one-way analysis of variance was used to evaluate differences among multiple groups with Gaussian distribution, and *p*-values determined using Tukey’s multiple comparisons test. A nonparametric Kruskal–Wallis test and a Dunn multiple comparisons test were used for data that did not show Gaussian distribution. Differences were considered to be statistically significant at *p* < 0.05.

## 3. Results

### 3.1. Transcriptomic Study

#### 3.1.1. Transcriptomic Changes Induced by VCR Exposure in Mouse DRG

**Figure 2:**
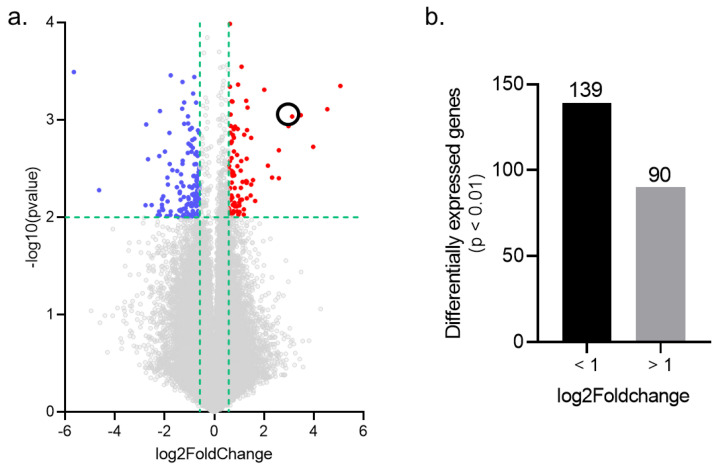
Analysis of differentially expressed genes (DEGs) in mouse DRG exposed to VCR. (**a**) Volcano plot of all dysregulated genes (*p* < 0.01, |FC| > 1.5). The most highly upregulated or downregulated genes are represented in red and blue, respectively. (**b**) VCR administration induced the upregulation of 90 genes and the downregulation of 139 genes in the DRG. The black circle indicates the *cck2r* gene. DRG: dorsal root ganglion.

Investigation of microarray gene expression was performed at D7 of VCR exposure in order to define gene expression changes during the induction of VIPN. The previously described mouse model of VIPN used mimics features observed in humans, i.e., mechanical allodynia of the lower limbs without any motor impairment associated with sensory nerve degeneration [19]. In total, 229 genes were found to be significantly differentially expressed in VCR compared to Ctrl mice (*p* < 0.01, |FC| > 1.5), of which 90 genes were upregulated and 139 were downregulated (Figure 2). 

Among the highly upregulated genes were *ucn*, *atf3*, *cck2r*, *sprr1a*, *ecel1* and *dync1h1*, which have all been associated with altered pain perception, neuropathic pain and nerve injury in previous studies (Table 2). Highly downregulated genes included *ms4a1*, *sema5a* and *bcl11a*, which have also been shown to be involved in nerve injury processes (Table 3). 

**Figure 3:**
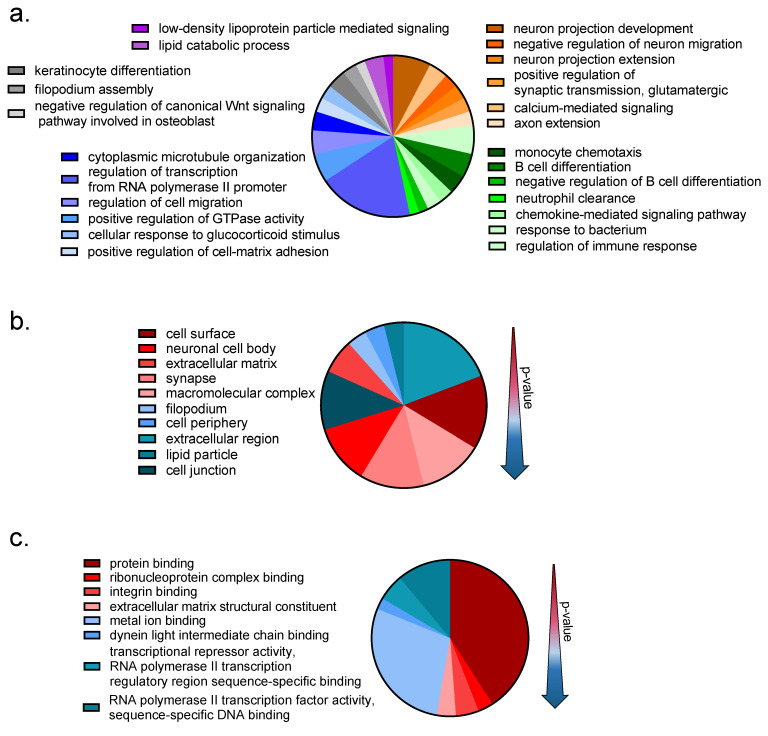
GO-enrichment analysis results obtained using DAVID database. All 229 differentially expressed genes (*p* < 0.01 and |FC| > 1.5) in the DRG following VCR exposure were submit to the DAVID Database in order to identify enriched GO terms associated with these gene list. (**a**) Biological processes. The darker color refers to lower *p*-values. (**b**) Cellular component. (**c**) Molecular function. Limits were applied to an enrichment score > 0.5 and *p* value < 0.05.

The GO-enrichment analysis was performed to elucidate the biological processes (BP), cellular components (CC) and molecular functions (MF) associated with the 229 DEG transcripts in DRG following VCR exposure. This global analysis showed a predominant effect of VCR treatment on genes associated with inflammatory and immune responses and the neuronal system (Figure 3).

The GO enrichment analysis (BP) focusing on upregulated genes highlighted 5 upregulated genes associated with “neuron projection development” (c*amsap2*, *lingo1*, *nrxn1*, *stmn4* and *ucn*), 3 downregulated genes associated with “negative regulation of neuron migration” (s*rgap2*, g*sk3* and *tnn*) and two genes associated with “positive regulation of synaptic transmission, GABAergic” (*cck2r*, *nrxn1*). Cellular components (GO-term) of the “neuronal cell body” were significantly upregulated (c*4b*, *dync1h1*, *nrxn*, *kcnj14*, *snap25* and u*cn*). The most significant GO-molecular functions associated with upregulated genes were “integrin binding”, “ribonucleoprotein complex binding” and “extracellular matrix structural constituent”. 

Significantly downregulated genes were associated with the following GO biological processes: “monocyte chemotaxis”, “positive regulation of GTPase activity” and “regulation of cell migration”. The GO cellular components associated with downregulated genes were “cell surface”, “macromolecular complex” and “nucleus”. The most significant GO molecular functions associated with downregulated genes were “integrin binding”, “ribonucleoprotein complex binding” and “extracellular matrix structural constituent”. 

#### 3.1.2. CCK2R mRNA Is Overexpressed in DRG of VCR-Administrated Mice

VCR exposure led to an upregulation of the expression of the *cck2r* gene in mouse DRG (FC = +8.75, *p* = 0.0009 VCR vs. Ctrl). Given the established implication of the entire cholecystokinin system, namely cholecystokinin and its two receptors, CCK1R and CCK2R, in pain modulation, we included the *cck* and *cck1r* genes in our search. However, their expression did not show a significant change following VCR exposure (Table 4).

**Figure 4:**
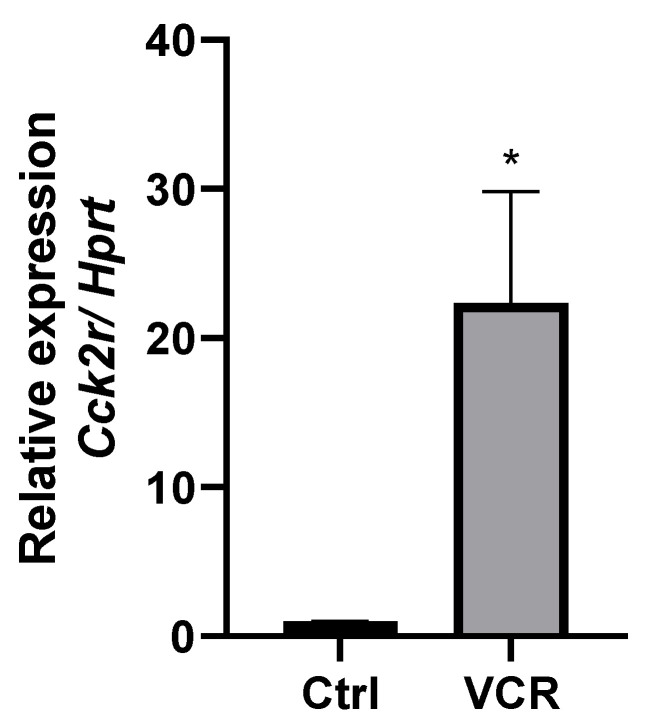
Relative expression of *cck2r* assessed by RT-qPCR. qPCR relative expression was calculated using the ΔCt method and normalized to *hprt* gene expression. * *p* < 0.05 VCR vs. Ctrl (n = 3 per group). Ctrl: control, VCR: vincristine.

CCK2R overexpression was further confirmed by RT-qPCR (Figure 4). 

#### 3.1.3. Location of CCK2R Protein in Mouse DRG and Sciatic Nerve

**Figure 5:**
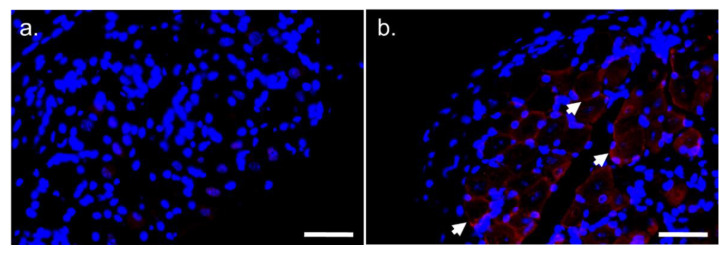
Expression of CCK2R protein in DRG of VCR mice assessed by immunofluorescence. Immunostaining of CCK2R on DRG from Ctrl mice (**a**) and VCR mice (**b**). White arrows indicate CCK2R staining. CCK2R: cholecystokinin type 2 receptor, Ctrl: control, DRG: dorsal root ganglia, VCR: vincristine. Scale bar = 0.05 mm.

To define the contribution of CCK2R to VCR pain modulation, the location of its distribution was assessed in the peripheral nervous system. The specificity of CCK2R antibody was first tested on the mouse stomach, where it is highly expressed in parietal and enterochromaffin-like cells (ECL). Interestingly, CCK2R staining could only be detected in the DRG of VCR mice (Figure 5) in the vicinity of neurons.

**Figure 6:**
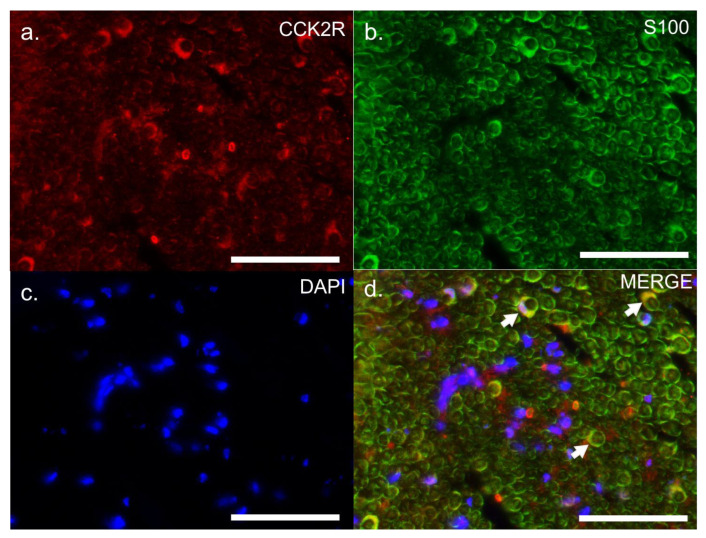
Dual labelling of CCK2R and S100 in the sciatic nerves of VCR-administrated mice. Dual labelling was carried out on sciatic nerves of VCR mice with antibodies directed against CCK2R (**a**) and S100 (**b**), a marker of Schwann cell cytoplasm. Nuclei were counterstained with DAPI (**c**). Co-labelling is indicated on the merged image by white arrows (**d**). CCK2R: cholecystokinin type 2 receptor, DAPI: 4′,6-diamidino-2-phenylindole, nuclear stain, S100: S100 β protein, VCR: vincristine. Scale bar = 0.05 mm.

**Figure 7:**
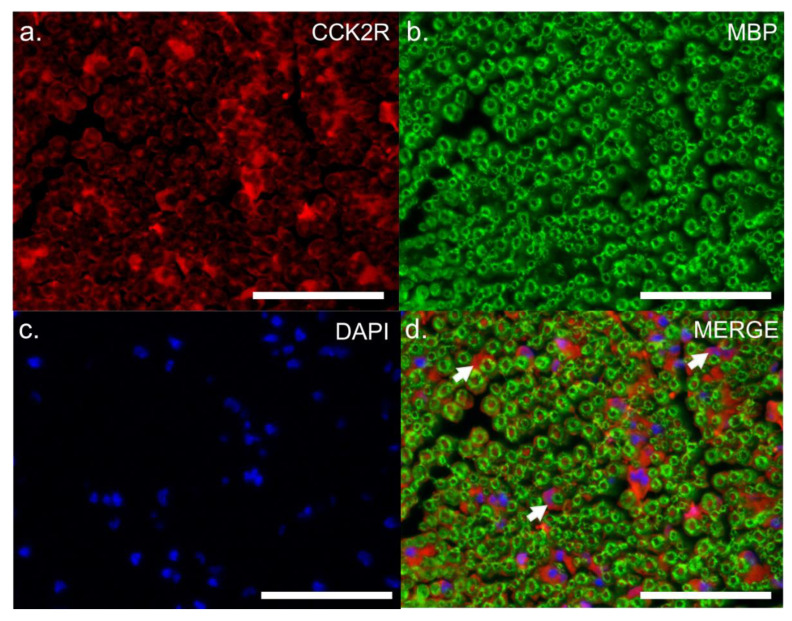
Dual labelling CCK2R and MBP in the sciatic nerves of VCR-administrated mice. Dual labelling was carried out on sciatic nerve sections of VCR mice with antibodies directed against CCK2R (**a**) and MBP (**b**), a marker of myelin sheath. Nuclei were counterstained with DAPI (**c**). On the merged image (**d**), CCK2R labelling was observed around MBP labelling (white arrow). CCK2R: cholecystokinin type 2 receptor, DAPI: 4′,6-diamidino-2-phenylindole, nuclear stain, MBP: myelin basic protein, VCR: vincristine Scale bar = 0.05 mm.

To gain insights into the precise location of CCK2R expression, we performed co-staining on sciatic nerve sections with S100, a cytoplasmic marker of adult Schwann cells (Figure 6), or with MBP, a myelin sheath marker (Figure 7). Co-localization of CCK2R and S100 was observed (Figure 6), whereas no dual labelling was observed with MBP (Figure 7), suggesting that CCK2R may be expressed more specifically in the cytoplasm of Schwann cells.

### 3.2. Pharmacological Study

#### 3.2.1. CCK2R Signaling Contributes to VCR-Induced Mechanical Allodynia

Two pharmacological tools, proglumide and Ly225910, were used to evaluate the relevance of CCK2R in VCR-induced mechanical allodynia observed in our mouse model. Proglumide, a non-selective antagonist of CCK1R and CCK2R, was used clinically for its analgesic properties in the 1980s [50,51,52] and is currently in a clinical trial (Phase I) for the management of nonalcoholic fatty liver disease (clinicaltrial.gov, NCT04152473). Ly225910 is a specific CCK2R antagonist thus far used only in research [53].

**Figure 8:**
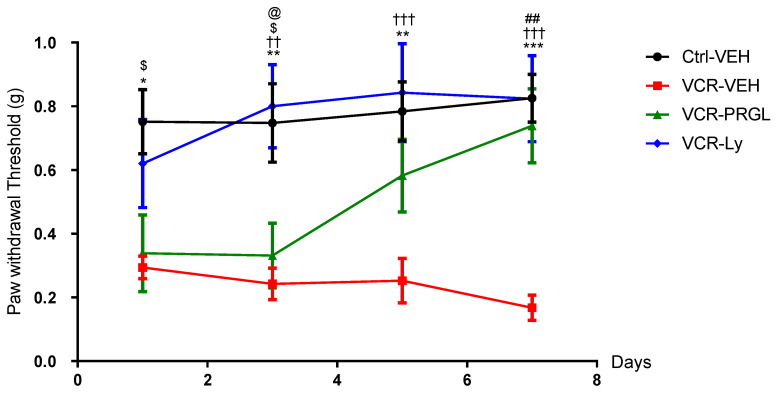
Effects of Ly225910 on mechanical allodynia induced by VCR in mice. Mechanical sensitivity was evaluated using the Von Frey filament test at D1 D3, D5 and D7. n = 8 mice per group, * *p* < 0.05, ** *p* < 0.01, *** *p* < 0.001, VCR-VEH vs. Ctrl-VEH. †† *p* < 0.01, ††† *p* < 0.001, VCR-VEH vs. VCR-Ly, $ *p* < 0.05 Ctrl-VEH vs. VCR-PRGL, ## *p* < 0.01 VCR-VEH vs. VCR-PRGL, @ *p* < 0.05 VCR-Ly vs. VCR-PRGL. Ctrl: control, Ly: Ly225910, PRGL: proglumide, VEH: vehicle, VCR: vincristine.

As described previously [19], mice in the VCR-VEH group developed a significant mechanical allodynia from D1 to D7 compared with mice in the Ctrl-VEH group (D1: *p* = 0.0146; D3: *p* = 0.0055; D5: *p* = 0.0031; D7: *p* = 0.0001). Proglumide, Ly225910 or vehicle (0.1% DMSO) had no significant effect on mechanical responses in the Ctrl groups at D1, D3, D5 or D7. VCR-administered mice treated with proglumide developed mechanical allodynia at D1, similar to that observed in the VCR-VEH mice. However, a significant difference was found between VCR-PRGL and Ctrl-VEH mice only at D1 and D3 (D1 *p* = 0.0339, D3 *p* = 0.0317) (Figure 8). PRGL accelerated the recovery of normal mechanical sensitivity in VCR-administered mice from D5 to D7, with complete restoration at D7 shown by a significant difference in mechanical threshold between VCR-PRGL and VCR-VEH groups (*p* = 0.0012) (Figure 8).

Although the trend was suggestive of a protective effect of Ly225910 on VCR-induced mechanical allodynia at D1, no significant difference in mechanical threshold was observed between VCR-VEH and VCR-Ly mice (*p* = 0.1349). However, Ly225910 treatment restored normal mechanical sensitivity, similar to that of Ctrl-VEH mice and significantly lower than that of VCR-VEH mice, from D3 to D7 (D3: *p* = 0.0017, D5: *p* = 0.0008, D7: *p* = 0.0002 VCR-VEH vs. VCR-Ly) (Figure 8).

#### 3.2.2. CCK2R Blockade Alleviates the Decrease in IENF and DRG Neuron Densities Induced by VCR

**Figure 9:**
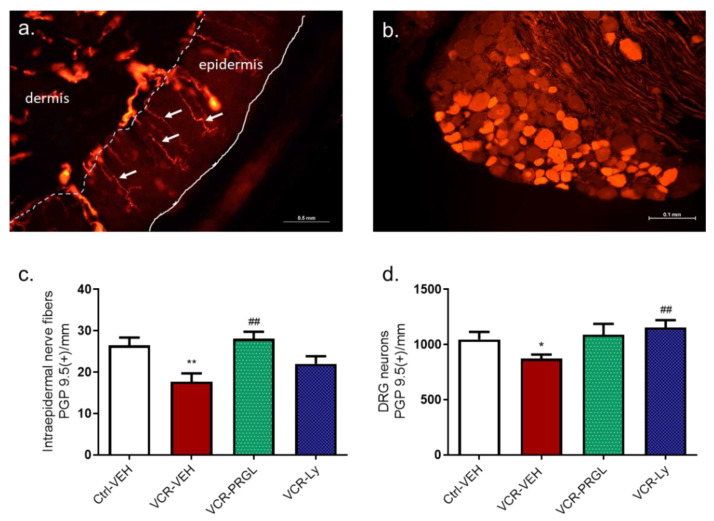
Effect of proglumide and Ly225910 on the loss of sensory nerve endings and neurons induced by VCR in mice. Immunohistochemistry for PGP9.5 was performed on paw skin sections (**a**) and DRG (**b**). (**c**) Intraepidermal nerve fiber density was assessed. Three sections of paw skin were examined per mouse. n = 6 mice. (**d**) DRG neuron density was quantified. Three DRG sections and three DRG per mice were counted. n = 6 per group. * *p* < 0.05, ** *p* < 0.01 vs. Ctrl-VEH. ## *p* < 0.05 vs. VCR-VEH. Ctrl: control, DRG: dorsal root ganglia, Ly: Ly225910, PGP9.5: protein gene product 9.5 PRGL: proglumide VEH: vehicle, VCR: vincristine.

The IENF density was lower in VCR-VEH mice than in Ctrl-VEH mice (*p* = 0.0043). Proglumide and Ly225910 had no effect on IENF and DRG neuron densities in the Ctrl groups. However, there was no significant difference in IENF density between Ctrl-VEH mice and VCR-VEH mice treated with PRGL or Ly225910 (Figure 9a). Similarly, the density of DRG neurons was significantly reduced by VCR administration (*p* = 0.0325 Ctrl-VEH vs. VCR-VEH). Treatment with proglumide and Ly225910 prevented the VCR-induced loss of DRG neurons (*p* = 0.0076, VCR-Ly vs. VCR-VEH) (Figure 9b).

#### 3.2.3. Effect of CCK2R Blockade on Alterations Induced by VCR on Myelinated Nerve Fiber Density and Morphology in Sciatic Nerves

**Figure 10:**
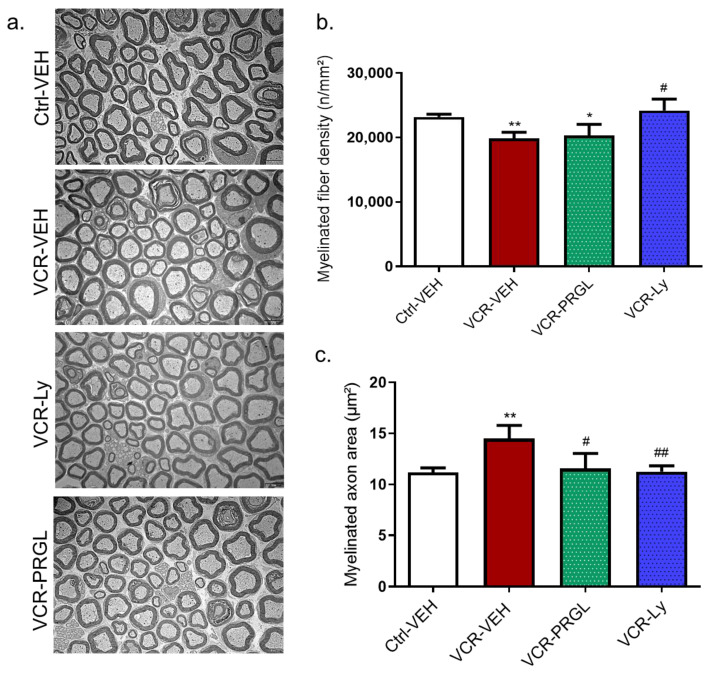
Quantitative analysis of sciatic nerves by electron microscopy. (**a**) Visualization of myelinated fibers in the sciatic nerve. (**b**) Quantification of myelinated fiber density. (**c**) Measure of myelinated axon area in the nerve. n = 6 per group; * *p* < 0.05, ** *p* < 0.01 vs. Ctrl-VEH. # *p* < 0.05; ## *p* < 0.01; vs. VCR-VEH. Ctrl: control, Ly: Ly225910, PRGL: proglumide, VEH: vehicle, VCR: vincristine.

Proglumide and Ly225910 did not affect the morphology of myelinated or unmyelinated nerve fibers in the sciatic nerves of the Ctrl groups; nevertheless, there were no notable changes in unmyelinated fiber morphology (Figure 10a) in VCR-VEH mice relative to Ctrl-VEH mice. Quantitative analysis of electron microscopy images showed that VCR treatment significantly decreased myelinated fiber density (*p* = 0.0076, Figure 10a,b). This decrease was associated with an increase in myelinated axon area (*p* < 0.01, Figure 10a,c). Proglumide and Ly225910 prevented the decrease in myelinated fiber density (*p* = 0.0325, Figure 10a) and the increase in myelinated axon area induced by VCR (*p* = 0.0043, Figure 10b), such that there was no significant difference between the myelinated axon areas of mice in the Ctrl-VEH, the VCR-Ly and VCR-PRGL groups (Figure 10a,c).

### 3.3. Pharmacological Modulation of CCK2R Did Not Alter the Anticancer Activity of VCR on Human Lymphoma Cell Lines

The influence of proglumide and Ly225910 on the anticancer activity of VCR was evaluated using two human diffuse large B-cell lymphoma cell lines (SU-DH-L4 and U-932) in agreement with VCR use in the clinic for these cancers. First, cell lines were exposed to VCR (from 0 to 2 nM) to assess its cytotoxic efficacy and to determine the [IC50] (1.065 nM for SU-DHL-4 and 0.959 nM for U-2932). 

**Figure 11:**
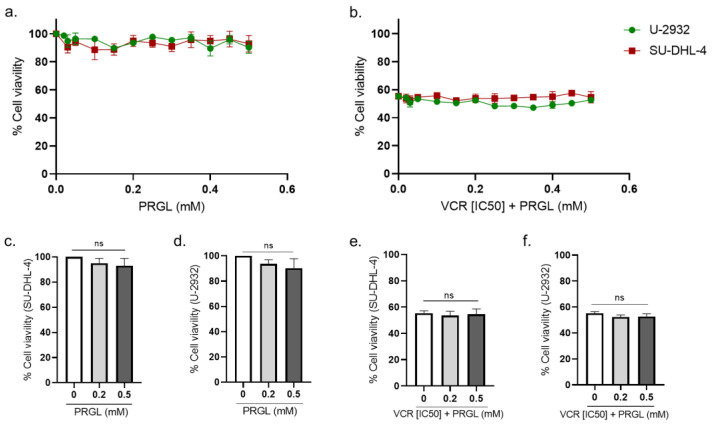
Effect of the combination of proglumide and VCR on the cell viability of human lymphoma cell lines. Cell viability was evaluated using MTT assay at 24 h on two lymphoma cell lines, SU-DHL-4 and U-2932. Cell viability was evaluated with proglumide alone (0.02–0.5 mM) (**a**,**c**,**d**) and with VCR at IC50 (**b**,**e**,**f**). ns = non-significant. IC50: half maximal inhibitory concentration, PRGL: proglumide, VCR: vincristine.

Proglumide alone had no effect on the cell viability of both cell lines (Figure 11a–c). In cells treated with VCR at [IC50], combination with proglumide did not significantly affect the anticancer activity of VCR in both cell lines (Figure 11d–f). 

**Figure 12:**
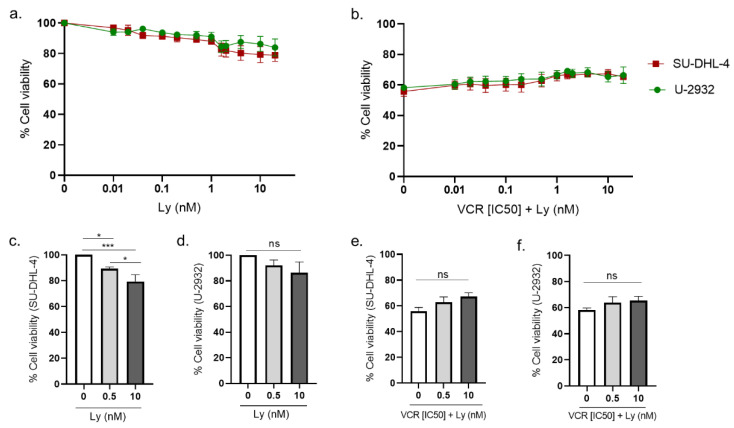
Effect of the combination of Ly225910 and VCR on cell viability of human lymphoma cell lines. Cell viability was evaluated by MTT assay at 24 h on two lymphoma cell lines, SU-DHL-4 and U-2932. Cell viability was evaluated with Ly225910 alone (0.001–10 nM) (**a**,**c**,**d**), and with VCR at IC50 (**b**,**e**,**f**). ns = non-significant, * *p* < 0.05, *** *p* < 0.001. IC50: half maximal inhibitory concentration, Ly: Ly225910, VCR: vincristine.

Ly225910 alone slightly reduced the viability of both cell lines (Figure 12a–c). In the SU-DHL-4 cell line, Ly225910 alone led to a significant decrease in cell viability of 10.81% at 0.5 nM (*p* = 0.0136, Ly (0.5 nM) vs. Ly (0 nM)) and of 20.67% at 10 nM (*p* = 0.0005, Ly (10 nM) vs. Ly (0 nM)) (Figure 12a,b). In the U-932 cell line, Ly225910 treatment reduced cell viability by 8% at 0.5 nM (*p* = 0.4985, vs. Ly (0 nM)) and by 14% at 10 nM, and this effect was not statistically significant (Figure 12a–c). 

In cells treated with VCR at (IC50), Ly225910 did not significantly affect the anticancer activity of VCR in both cell lines (Figure 12d–f), suggesting that the CCK2R blockade does not interfere with VCR efficacy.

## 4. Discussion

The main findings of this study were that (1) CCK2R is overexpressed, at both the mRNA and protein level, in mouse DRG following VCR exposure; (2) the blockade of CCK2R by proglumide or Ly225910 is an effective strategy to reduce mechanical allodynia and prevent sensory nerve fiber damage induced by VCR; and (3) proglumide or Ly225910 in combination with VCR did not adversely affect the anticancer activity of VCR in human cancer cell lines. Moreover, our immunofluorescence study showed that CCK2R appeared to be mainly expressed by glial cells, such as Schwann cells and satellite glial cells, in the peripheral nervous system. 

Our understanding of chemotherapy-induced peripheral neuropathy is complicated by its multifactorial underlying components, including the chemotherapy agent used and the heterogeneity of neuropathic symptoms. Transcriptomic analysis has great potential as an approach to identify potential gene regulatory processes and molecular details that could contribute to pain and nerve injuries in patients. Here, we aimed to investigate the potential changes in pathways following VCR exposure. Numerous genes involved in nerve regeneration, axonal projection or synaptogenesis were found to be upregulated in mouse DRG in response to VCR, whereas some genes associated with negative regulation of neuron migration were downregulated. The GO-enrichment analysis highlighted that the immune response-associated genes were significantly differentially expressed in the DRG following VCR exposure. These results are in line with those of Starobova et al., 2020, who reported that the regulatory genes associated with the immune system, but not those associated with the neuronal system, were affected by VCR treatment (40 µg/kg), suggesting the involvement of a neuroinflammatory mechanism [54]. These authors also reported that two genes that are implicated in axonal regeneration, *Atf3* and S*prr1a*, were highly upregulated following VCR exposure. These two genes are expressed only after a peripheral nerve injury and were also upregulated in our model [27,29]. Under our experimental conditions, treatment with VCR at a cumulative dose of 800 µg/kg affected the expression of genes associated with the immune response and the neuronal system, suggesting a combination of neuroinflammatory and neuropathic mechanisms being involved as a result of VCR neurotoxicity [55].

This preliminary screening by transcriptomic analysis also confirmed our interest in CCK2R as a potential therapeutic target for preventing VIPN. Indeed, the overexpression of C*ck2r* is noteworthy for several reasons. First, CCK2R is a G-protein-coupled receptor, a major class of proteins that represent a preferred therapeutic target for novel analgesics [9,56]. Second, several studies have demonstrated the involvement of CCK2R in the modulation of nociception, notably by central mechanisms [9,57,58]. Finally, several CCK2R antagonists have been developed, and some of these have been shown to successfully alleviate pain in other rodent models [57,59].

CCK2R is preferentially expressed in the gastrointestinal tract and central nervous system and is well known for its role in satiety, anxiety and pain [11,12]. Under physiological conditions, CCK2R is expressed at a very low level in the peripheral nervous system, but this expression may be dramatically increased in pathological states [15,17,57]. Our immunofluorescence results showed that CCK2R is expressed at a very low level in the DRG under physiological conditions but is overexpressed in the case of VCR exposure. In addition, overexpression of CCK2R in the DRG has been reported in a rat model of diabetes, suggesting its possible involvement in healing mechanisms after toxic nerve injury [60]. Moreover, CCK2R seems to be preferentially expressed in glial cells, such as satellite glial cells and Schwann cells, cell types well known for their role in the maintenance and repair of peripheral nerves. Thus, the localization and regulation of CCK2R support its possible involvement in neuroprotection/neuro-regeneration processes.

Subsequently, based on our transcriptomic/morphological results and the literature, we took a pharmacological approach to block CCK2R in the mouse model of VIPN. Daily administration of proglumide did not prevent the onset of mechanical allodynia induced by VCR but did accelerate the recovery of normal mechanical sensitivity in VCR-administered mice. Recently, it has been shown that proglumide reduced mechanical allodynia in a rat model of burn-induced pain [15]. Analgesic effects of proglumide have already been described in other preclinical and human pain models, such as post-operative pain, burn-induced pain and neuropathic pain. In these reports, proglumide was administered alone or in combination with morphine or other opioids. Even though some studies showed negative effects [61,62,63], the co-administration of proglumide with opioids generally potentiated its anti-nociceptive effect and prevented morphine tolerance [50,51,52,64,65]. The interaction of CCK/morphine systems takes place at the supraspinal, spinal and peripheral level, as has been highlighted in a diabetic rat model [66]. Under our experimental conditions, proglumide alone showed analgesic properties on VCR-induced mechanical pain, as has been previously shown in the burn-induced pain model cited above [15]. 

Proglumide is a nonselective antagonist of CCK1R and CCK2R. CCK2R is the most highly expressed receptor in the mouse nervous system [67] and was also overexpressed in the DRG of VCR-exposed mice. Thus, in order to evaluate the specific effect of CCK2R blockade, mice exposed to VCR were treated with Ly225910. Pretreatment with Ly225910 totally prevented the onset of VCR associated-mechanical allodynia. Several studies have already demonstrated that the blockade of CCK2R is beneficial for pain treatment [9,57,58]. A study in CCK2R-deficient mice suggested that mechanical sensitivity as well as the development of neuropathic pain are regulated by antagonistic interactions between CCK receptors and opioid receptors. Indeed, CCK2R is able to dimerize with the opioid receptor MOR [12]. Once activated, CCK2R forms heterodimers with MOR, preventing the binding of opioids to their receptor, thus blocking their analgesic effect. In addition, CCK2R can inhibit transient type-A K^+^ channels [68]. In DRG neurons, activation of CCK2R by its ligand, CCK-8, induced a decrease in type-A K^+^ currents and involved the PI3K/SRC/JNK pathway. Voltage-dependent potassium channels (Kv) play a major role in the excitability of primary sensory neurons by promoting the repolarization step during the generation of action potentials. The decrease in type A K^+^ current increases neuronal excitability [69] and, in this way, leads to hypersensitivity and thermal hyperalgesia [70,71,72,73]. A reduction in Kv activity is associated with an increase in the frequency and duration of action potentials resulting in membrane hyperexcitability [74]. Therefore, specific blockade of CCK2R could inhibit this reduction in the K^+^ current and DRG neuron hyperexcitability and thus reduce pain.

Functional results in this study were reinforced by histological analysis. Both proglumide and Ly225910 were able to block all neuronal damages caused by VCR neurotoxicity. The loss of IENF, the decrease in myelinated fiber density and the swelling in myelinated fibers were all inhibited by both proglumide and Ly225910. Therefore, under our experimental conditions, the inhibition of the CCK system by proglumide and the specific blockade of CCK2R by Ly225910 showed analgesic properties, in agreement with observations made in other neuropathic pain models. The neuroprotective effects obtained in our model have not been previously described to our knowledge. Several hypotheses could explain this neuroprotective/neuro-regenerative effect. Activation of microglia and astrocytes in the spinal cord during VCR exposure has been suggested to lead to cytokine release, thus maintaining neuropathic pain by inflammatory mechanisms [75,76]. The blockade of the CCK system may be anti-inflammatory, preventing neuroinflammation and consequently hyperexcitability of the sensory nervous system induced by VCR exposure. This anti-inflammatory effect of CCK blockade has already been shown in a model of chronic pancreatitis treated with proglumide [77]. CCK2R is a G-coupled protein predominantly coupled with the Gq protein. Activation of Gq results in a massive Ca^++^ efflux from intracellular calcium endoplasmic reticulum stores, leading to increased neuron excitability. Overall activation of CCK2R, and thus rapid and repeated mobilization of intracellular Ca^++^, contributes to excitotoxic conditions and neuronal degeneration [78]. The blockade of CCK2R, which is upregulated in the DRG by VCR, may protect against this excitotoxicity.

In addition to analgesic and neuroprotective properties, the use of proglumide and Ly225910 did not seem to alter the anticancer activity of VCR. Indeed, in vitro experiments performed on human lymphoma cell lines treated with VCR showed that simultaneous administration of proglumide or Ly225910 with VCR did not reduce the cytotoxicity of VCR. Ly225910 induced a light but significative decrease of the viability of both human cell lines used. However, this effect was lost in combination with VCR, and there was no significative alteration of the VCR cytotoxicity. Thus, these results obtained on human cell lines have to be interpreted with caution, and further investigations on other human cell lines and with different techniques should be performed. In addition, further studies on VCR-treated animals bearing cancers are also required to confirm if these effects are reproductible in vivo.

In addition to proglumide, and in the perspective of drug repurposing, several more selective CCK2R antagonists have recently emerged and may be good candidates to prevent VIPN. Among these, netazepide (Trio Medecine Ltd., London, UK), or YF476, is well tolerated in humans [79] and has been the subject of several clinical trials, notably in the management of gastric neuroendocrine tumors (clinicaltrial.gov NCT01339169) [80]. In conclusion, targeting CCK2R may represent an effective strategy for preventing the onset of VCR-induced neuropathic pain, thus improving both patient quality of life and rates of cancer survival.

## Figures and Tables

**Table 1:** Primer sequences used for real-time PCR.

Genes (Size)	Primers (5′-3′)	Accession No.
Mouse *cck2r* (122 bp)	Designed by Qiagen (QuantiTect Primer assay)	NM_007627
Mouse *hprt* (185 bp)	F: TGATCAGTCAACGGGGGACAT R: AGGTCCTTTTCACCAGCAAGC	NM_013556.2

**Table 2:** List of highly upregulated genes (*p* < 0.01, |FC| > 1.5) in the DRG of mice exposed to VCR compared with Ctrl mice.

Gene Symbol	Description	*p*-Value	Up FC	Ref.
*ucn*	Urocortin (Ucn), transcript variant 1	4.42 × 10^−4^	33.59	Oxidative stress, neuroprotective in Parkinson’s disease [26] repair
*sprr1A*	Small proline-rich protein 1A	7.75 × 10^−4^	23.35	Promotes axonal outgrowth [27]
*dync1h1*	Dynein cytoplasmic 1 heavy chain 1	0.0018	15.76	The main motor protein responsible for retrograde axonal transport in neurons [28]
*atf3*	Activating transcription factor 3	0.0020	6.06	Survival and the regeneration of axons following axotomy [29]
*ecel1*	Endothelin converting enzyme-like 1	0.0039	6.05	Nerve development, nerve regeneration [30]
*mill2*	MHC I like leukocyte 2, transcript variant 1	0.0029	4.45	Expressed by Schwann cells, involved in antigen presentation [31]
*lingo1*	Leucin rich repeated and ig domain containing 1	0.0033	2.08	Negatively regulates myelination by oligodendrocytes [32]
*stmn4*	Stathmin-like 4	0.0093	1.95	Marker for polyneuropathy in primary Sjögren’s syndrome [33]
*snap25*	Synaptosomal-associated protein 25	0.0086	1.70	Cell exocytosis during synaptic transmission. Cleaved by Botulinum toxin, implicated in neuropathic pain [34]
*MMP16*	Matrix metallopeptidase 16	0.0014	1.68	Regulates neuronal responsiveness to myelin [35]
*ptprd*	Protein tyrosine phosphatase, receptor type, D	0.0098	1.64	Increased in DRG, neuropathic pain in CCI rat and mice [36,37]
*nwd1*	NACHT and WD repeat domain containing 1	0.0043	1.62	Facilitates synaptogenesis in spinal cord and then neuropathic pain [38]
*sumo2*	Small ubiquitin-like modifier 2	0.0017	1.60	Overexpressed after nerve injury, role in regeneration [39]
*camsap2*	Calmodulin regulated spectrin-associated protein family, member 2	0.0065	1.58	Control of dendritic microtubule organization [40]
*flt3*	FMS-like tyrosine kinase 3	0.0032	1.51	Alleviates pain if downregulated [41]

Ctrl: control, DRG: dorsal root ganglion, VCR: vincristine.

**Table 3:** List of highly downregulated genes (*p* < 0.01, |FC| > 1.5) in the DRG of mice exposed to VCR compared with Ctrl mice.

Gene Symbol	Description	*p*-Value	Down FC	Ref.
*ms4a1*	Membrane-spanning 4-domains, subfamily A, member 1	0.0013	24.72	B-lymphocyte antigen (CD20)
*cyp2e1*	Cytochrome P450, family 2, subfamily e, polypeptide 1	0.0079	4.28	Regulates response to oxidative stress [42]
*sema5a*	Semaphorin 5A	0.0097	3.75	Schwann cell injury response [43], axon guidance molecule in the nervous system [44]
*gabrg3*	Gamma-aminobutyric acid (GABA) A receptor, subunit gamma 3	0.0066	2.55	Synaptogenesis, nociception [45]
*cp*	Ceruloplasmin	0.0098	1.86	Provide iron to axonal mitochondria [46]
*bcl11a*	B-Cell Lymphoma/Leukemia 11A	6.63 × 10^−4^	1.66	Negative regulation of dendrite extension [47]
*dmd*	dystrophin, muscular dystrophy	0.0053	1.61	Synaptic transmission [48]
*prox1*	Prospero Homeobox 1	0.0087	4.63	Induces new lymphatic vessel formation and promotes nerve reconstruction [49]

Ctrl: control, DRG: dorsal root ganglion, VCR: vincristine.

**Table 4:** Expression of genes related to CCK2R during VIPN induction.

Gene	VCR	
	FC	*p*-Value
*cck2r*	8.75	9.21 × 10^−4^ ****
*cck1r*	1.2	0.15
*cck*	10.6	0.78

CCK2R: cholecystokinin type 2 receptor, FC: fold change, VIPN: vincristine-induced peripheral neuropathy. **** *p* < 0.0001 VCR vs. Ctrl (n = 4 per group). Ctrl: control, VCR: vincristine.

## Data Availability

Not applicable.
